# Technique and maximal skiing speed for youth cross-country skiing performance

**DOI:** 10.3389/fspor.2023.1133777

**Published:** 2023-04-21

**Authors:** Roland Stöggl, Erich Müller, Thomas Stöggl

**Affiliations:** ^1^ Department of Sport and Exercise Science, University of Salzburg, Salzburg, Austria; ^2^ Bundesgymnasium/ Sportrealgymnasium (HIB), Saalfelden, Austria; ^3^ Red Bull Athlete Performance Center, Salzburg, Austria

**Keywords:** youth, cross-country skiing, talent development, skiing techniques, skiing performance, skiing speed, diagnostics, physical literacy

## Abstract

**Introduction:**

Numerous researches concentrate on examining and preparing high-level male cross-country skiers, with a significant number of tests being conducted on roller skis. However, there is a scarcity of research on the testing and preparation of younger male and female athletes ranging from 10 to 16 years old. The main purpose of this research was to determine if certain cross-country (XC) skiing tests and maturity status are indicators of performance in youth cross-country skiing; to examine any differences in performance between young males and females; and to establish non-invasive diagnostic tools for assessing performance.

**Methods:**

Fifty-eight young XC skiers (36 boys; 12.88 ± 1.19 yrs and 22 girls; 12.79 ± 1.09 yrs) performed specific XC skiing maximal speed tests consisting of short (50 m) flat and uphill distances (30/40 m). Results were correlated with on snow XC skiing performance (P_XC_) based on one skating (including an agility parcours) and one classical distance competition.

**Results:**

The key findings of this research were: 1) Age and maturity status were associated to boys'and girls' P_XC_; 2) Significant moderate to high correlations between girls' and boys' short duration XC skiing sprint performance 30-50 m (double poling (DP) flat and uphill, free skating, leg skating and V1 uphill skating) and P_XC_ were revealed; 3) In general, the best prediction for P_XC_ (Boys and Girls) was found to be the asymmetrical uphill (V1 40 m uphill) sub-technique; and 50 m DP (flat) while Boys' P_XC_ was determined by V1 skating and girls' performance mainly by 50 m free skating (flat); 4) When using maturity offset as a confounding variable, boys' and girls' P_XC_ was still highly associated with short duration skiing tests.

**Discussion:**

In conclusion, the use of simple, non-invasive XC skiing sprint tests for evaluating P_XC_ can be beneficial for ski clubs, specialized schools, or skiing federations in identifying and training young talented skiers. Further, this result demonstrates that skiing abilities such as short duration maximal speed and the proper use of different sub-techniques at high speeds during XC skiing is an important performance prerequisite.

## Introduction

It is crucial that any physical training that young athletes engage in is appropriately structured and supervised. This means that coaches and trainers must have the appropriate knowledge, skills, and qualifications to design and implement safe and effective training programs for young athletes (1–3). They should also be familiar with the principles of long-term athletic development and be able to adapt the training program as the athletes grow and develop (4, 5). This means that the training and conditioning of young athletes must be tailored to their age, skill level, and stage of development (1, 2, 6–8).

To achieve elite status in most sports, it is recommended to specialize late and diversify early in training. Furthermore, athletes who start sport-specific training at a young age tend to have shorter athletic careers (9).

In this context, cross-country (XC) skiing abilities and a wide range of motor abilities should also be developed with a wide focus on versatile training solutions in younger ages (10, 11).

Recent studies on elite adult XC skiers have shown a significant shift in the emphasis on force and speed components in determining performance. These studies found that factors such as maximal skiing speeds, explosive or maximum force, and muscular endurance power were also important predictors of XC skiing performance. All these findings are reported in a systematic review of the effects of strength and power training on performance in XC skiers (12).

Competitive XC skiing today requires a highly dynamic and complex full-body motion, where skiers face a wide range of speeds and constantly changing terrain, requiring varying levels of effort and frequent shifts between different subtechniques at high speeds (13–17). To perform at the highest level in these specialized disciplines, athletes must improve their physiological, technical, and tactical abilities (13–17).

Stöggl et al. (10) found that as skiing speeds and techniques have evolved, the importance of speed, strength, and coordination has increased for young XC skiers.

It is likely that this advancement is due to the better training and testing methods being used in this age group and highlights the significance of utilizing high-quality training and testing processes for young skiers (10).

Many studies focus on the testing and training of elite male XC skiers, but very little research is available on the testing and training of younger athletes aged between 10 and 16 years (10, 11).

It is worth noting here that an outstanding share of the mentioned maximal speed skiing tests (14, 18–21) was performed on roller skis emphasizing the need for on-snow XC skiing tests.

Sollie et al. (22) attempted to investigate the differences in pacing and technique selection between young male skiers (14.4 ± 0.5 years) and competitive adult skiers and found that variations in physical ability affect speed and technique choice, emphasizing the need for age-specific technical training for different skill levels.

In addition, it is difficult to find any research that validates XC skiing ability tests in relation to the performance of young XC skiers. Furthermore, information scarce on elite female and young female XC skiers in particular. Only a handful of studies, four specifically, have included young female athletes in their research (23–26).

As far as we know, our research group's studies are the only ones that have linked validation of motor ability tests, anthropometrics, and roller skiing speed tests to the performance of young XC skiers (girls and boys aged 12.5–14 years.) (10, 11) concluding the need for further investigations on snow.

The changes and increased demands in adult elite XC skiing, such as higher skiing speeds and changes in skiing techniques with a greater emphasis on explosive strength and upper body, along with the lack of research on young male and female skiers, highlight the importance of conducting performance evaluations to examine the relationships between short-duration XC skiing tests and XC skiing performance index (P_XC_) of young male and female skiers. This can aid in the training and competition process. Therefore, the current study aimed to (1) investigate whether tests specific to on-snow XC skiing, including maximum skiing speed, predict performance in young athletes and (2) examine the impact of maturation status on performance in young skiers and investigate whether there are any differences between male and female skiers.

## Methods

### Participants

This study included 58 young XC skiers, 36 males and 22 females, who volunteered to participate. The participants were selected based on two criteria: (a) attending a specialized school for XC skiing or being a member of a regional XC skiing team and (b) having at least 2 years of experience in training and competing in XC skiing. According to McKay et al. (27), the young participants, including top-performing athletes at the regional level, as well as winners in Austrian National Championships, were considered to be Tier 2 (trained/developmental) to Tier 3 (highly trained/national level). The participants' characteristics are summarized in Table 1.

**Table 1:** Age and anthropometrics, lat test (1RM).

	Boys (*n* = 36)					Girls (*n* = 22)					Diff. %	*p*-values
	Mean (±SD)	CI-L	CI-U	MIN	MAX	Mean (±SD)	CI-L	CI-U	MIN	MAX		
Age (years)	12.88 (1.19)	12.21	13.25	10.67	15.14	12.79 ± (1.09)	12.39	13.19	10.92	14.98	−0.7	0.9
Body height (cm)	164.0 (9.8)	160.9	167.1	142.0	181.0	161.8 (7.0)	159.3	164.3	144.0	175.0	−1.2	0.39
Body mass (kg)	51.3 (9.6)	48.2	54.3	34.0	68.5	49.8 (8.2)	46.9	52.8	34.2	67.0	−2.8	0.44
Sum of skinfolds (mm)	42.6 (12.4)	38.2	46.9	25.0	75.5	49.1 (14.2)	43.9	54.2	25.5	80.0	15.3	<0.001
Subischial leg length (cm)	80.5 (5.0)	78.8	82.3	70.0	89.5	78.4 (3.9)	76.9	79.9	69.5	87.5	−2.8	0.13
Sitting height (cm)	83.7 (6.3)	81.6	85.9	72.0	101.0	83.7 (4.2)	82.1	85.3	74.5	92.0	0.0	0.88
APHV (years)	13.37 (0.63)	13.15	13.59	11.61	14.42	11.76 (0.42)	11.60	11.92	10.7	12.49	−12.1	<0.001
Maturity offset (years)	−0.62 (1.19)	−1.04	−0.2	−2.33	1.76	0.92 (0.94)	0.56	1.28	−1.12	2.89	−248.3	<0.001
Lat 1RM	39.5 (5.9)	37.2	41.8	30.0	50.0	36.5 (8.2)	32.9	40.3	22.5	55.0	−7.4	0.18
Lat 1RM % body weight	81.6 (10.2)	77.6	85.6	64.2	101.8	74.4 (13.2)	68.4	80.3	55.2	96.6	−8.9	<.05

CI-L/CI-U, confidence interval lower and upper bound 95%; SD, standard deviation; APHV, estimated peak height velocity; Diff. %, difference of girls vs. boys; 1RM, one-repetition maximum.

All participants in the study willingly agreed to participate and were informed about the study purpose and procedures, and their parents or legal guardians provided written informed consent. The study was carried out in compliance with the Declaration of Helsinki and received ethical clearance from the local ethics committee of the University of Salzburg .

### Overall study design

Each individual participant underwent various noninvasive XC skiing tests and had their anthropometric data measured. All XC skiing tests were conducted on the same day and at least 2 weeks prior to the chosen competition.

Previous research has shown that the maximal speed skiing tests chosen for this study have a high level of reliability (*r* > 0.98) and validity when compared to the simulated sprint test performance of elite junior and adult XC skiers [1,000-m double poling (DP) and 1,100-m classical sprint: *r* > 0.85] (19).

Similar tests have been utilized in previous international testing protocols for adult XC skiers prior to the current study.

### Test protocols

A total of seven tests were performed in 1 day.

Before the start, all participants completed their individual usual competition warm-up routine (30 min) beside the starting area and on the track using their poles and equally prepared XC-Skating and Classic Skis by a service team.

#### XC skiing sprints (flat)

Short-distance sprint performances in different techniques [DP (50 m), free-technique (F 50 m), and leg skating (L 50 m)] were measured through 50-m sprint tests on skating skis. The tests were conducted on a flat, straight XC skiing track. Participants completed three trials for each technique, with the testing order and recovery time between the tests controlled by bib numbers starting from one. The results of the best trial for each technique were used for analysis. The starting position was standardized by positioning the ski with the binding 50 cm before the starting line and placing the poles on the ground. Time measurement started when the athlete passed the first light sensor at the starting line and ended when they passed the 50-m mark.

#### XC skiing sprint (uphill)

The athletes were required to complete an uphill distance of 30-m DP (30 m) and the asymmetrical uphill 40-m V1 skating (XC skiers have a dominant and a nondominant side) with a slight incline of 0–1° for the first 5 m and an incline of 4°–5° for the remaining distance.

#### 1,160-m classic test competition

Athletes started (individual start intervals of 30 s) at the lowest point of the track avoiding one longer downhill passage at the beginning of the track. Getting to the start accompanied by a team of coaches was used as a further specific warm-up and getting used to the “grip waxed” skis. After an uphill climb of 400 m, an intermediate time was taken.

#### 3,000-m skating competition (obstacle and agility parcours)

The agility course contained several demanding sections passed by all athletes. In between the agility parts, athletes had to use appropriate skating techniques adapted to the terrain for some longer passages also including uphill sections. After the individual start (start intervals of 30 s), each competitor had to skate a marked eight followed by slipping under several skiing gates, passing three waves and a slalom as well as a small jump and four giant slalom gates. The overall course (1,500 m) had to be passed twice.

The overall skiing performance index (P_XC_) was calculated by normalizing the skiing times of both races (3,000 m skating including parcours and 1,160-m classic) using *z*-standardization [by using equation $(x_{i}-\bar{x})/s$] and then taking the average of the two resulting *z*-values, which represents P_XC_. The two competitions selected were chosen based on consistent weather and snow conditions, individual start intervals, and the full cooperation of the participants.

#### One-repetition maximum lat pull-down test (cable lat pull-down)

To evaluate the maximal force of the upper body (target muscles: latissimus dorsi, trapezius pars ascendens, rhomboideus minor et major, teres major, and supporting muscles like erector spinae, biceps brachii, brachialis, and brachioradialis) 5 days prior to the XC skiing sprints, a one-repetition maximum (1RM) lat pull-down test was performed using a machine with a seated position and support across the top of the quadriceps. Participants began with a 10-repetition warm-up using a weight of 30%–50% of their estimated 1RM. For the 1RM test, the seat was adjusted to ensure full arm extension during the eccentric phase of the exercise, and the seat height was adjusted to allow the participants to start the lift with their arms fully extended. A pronated wide overhand grip (hands slightly wider than shoulder width, palms facing away from the body) was used throughout the test.

For the attempt to be counted, the bar had to be pulled down to the participant's chin while maintaining an upright posture. The motion should be smooth; sudden jerks, hunching, or swinging the body was not allowed. Participants were able to select their starting weight based on past experience. If the attempt was successful, the weight was increased by 1.0–2.5 kg, depending on how easily they could complete a single repetition. If the attempt was unsuccessful, the weight was reduced, and another attempt was given. A minimum of 5 min of recovery was given between attempts. This process was repeated until a complete repetition was not possible (28, 29). This testing method has been found to have a high level of reliability, with ICCs ranging from 0.979*** (male) to 0.998*** (female) to 0.995* (total) (30).

#### Assessment of anthropometrics and maturity

Anthropometric data including body stature, body mass, inseam leg length, and sitting height were collected. In addition, the sum of skinfold (mm) was calculated by the four-site caliper skinfold method: triceps, biceps, subscapular, and suprailiac (31).

To account for any potential effects of maturation, age from peak height velocity (PHV) and the maturity offset were calculated using chronological age, body height, sitting height, and body mass according to the method described by Mirwald et al. (32).

### Statistical analyses

All data were normally distributed, as confirmed by the Shapiro–Wilk test, and presented as means and standard deviations (±SDs). The raw data for each race were standardized using *z*-scores, and the mean across both competitions (1,160-m classic and 3,000-m skating) was taken to calculate P_XC_. The distances of the XC skiing competitions were equal for girls and boys. To investigate the relationship between test performance and P_XC_, Pearson's product moment correlations (*r*_xy_) were calculated. To account for multiple comparisons, the Bonferroni–Holm step down correction was applied. In addition, partial correlations (*r*_xy-z_) with maturity offset as a confounding variable were calculated in cases where there was a correlation between age or estimated maturity status and P_XC_. Additionally, variables that were significantly correlated to P_XC_ were analyzed through stepwise multiple regression to determine the most significant factors contributing to P_XC_. Sex differences in roller skiing results and anthropometric variables were established using *t*-tests for independent samples. The correlation values are classified as follows: excellent, 0.9–1.0; high, 0.8–0.9; moderate, 0.7–0.8; acceptable, 0.6–0.7; and low, <0.6. The significance level was set at *α* < 0.05. All statistical tests were performed using SPSS 28.0 Software (SPSS Inc., Chicago, IL, United States) and Office Excel 2021 (Microsoft Corporation, Redmond, WA, United States).

## Results

### Race performance

Distances and finishing times for girls' skating (3,000 m, 656.0 ± 291.6 s) and classic competition (1,160 m, 359.6 ± 50.3 s) were not different from those for boys' skating (3,000 m, 630.7 ± 300.7 s) and classic competition (1,160 m, 362.1 ± 50.1 s).

### Anthropometrics, age and maturity status, and 1RM lat pull-down test

For both female and male youths, age and anthropometrics, such as height, sitting height, and maturity offset, revealed low to moderate correlations with P_XC_ (*r* = −0.52 to −0.76, all *p*’s < 0.05 to <0.001).

Only girl's 1RM lat pull-down test was related to XC skiing performance (*r* = −0.53, *p* < 0.05) (Table 2).

**Table 2:** Bonferroni–Holm adjusted correlations (*r*_xy_) between calendar age, anthropometric data, 1RM lat test, and P_XC_ (s).

	Boys (*n* = 36)	Girls (*n* = 22)	All (*n* = 58)
	*r*_xy_	*r*_xy_	*r*_xy_
Age (years)	−.64^***^	−.70^***^	−.65^***^
Body height (cm)	−.52^**^	−.53^**^	−.52^**^
Sum of skinfolds (mm)	.15	−.21	−.04
Body weight (kg)	−.31	−.56^**^	−.40^**^
Subischial leg length (cm)	−.59^**^	−.34	−.51^***^
Sitting height (cm)	−.56^**^	−.64^***^	−.56^**^
APHV (years)	−.15	−.31	−.19
Maturity offset (years)	−.71^***^	−.76^***^	−.52^***^
Lat 1 RM	.20	−.53^*^	.22
Lat 1 RM perc. body weight	−.40	−.10	.20

APHV, estimated peak height velocity; 1RM, one-repetition maximum; P_XC_, XC skiing performance index.

^*^ *p* < 0.05. ^**^p < 0.01. ^***^p < 0.001.

### XC skiing tests

Table 3 shows detailed information about the times and speeds of the various XC skiing tests. Table 4 displays the correlations between P_XC_ and variables of the individual tests. The results show that for both boys and girls, there are high correlations between P_XC_ and V1 40-m uphill and free skating 50-m (*r* = 0.85 to 0.89, all *p*’s < 0.001 < 0.01).

**Table 3:** Sex comparison in XC skiing performance.

		Boys (*n* = 36)					Girls (*n* = 22)					Diff. %	*p-*values
		Mean (±SD)	CI-L	CI-U	MIN	MAX	Mean (±SD)	CI-L	CI-U	MIN	MAX		
Times (s)	V140-m uphill	10.34 (1.01)	9.94	10.74	8.0	12.07	10.97 (1.33)	10.35	11.58	8.37	13.64	6.1	0.9
	DP 30-m uphill	9.35 (1.13)	8.98	9.73	7.30	11.84	9.55 (1.29)	9.01	10.09	7.43	13.4	2.1	2.1
	Free skating 50-m	9.16 (.79)	8.90	9.42	7.23	10.32	9.50 (.71)	9.21	9.80	8.11	10.58	3.7	3.7
	DP 50-m	10.73 (1.11)	11.37	12.10	9.02	13.91	11.95 (1.22)	11.44	12.46	9.72	14.12	1.8	1.8
	Leg skating 50-m	9.56 (.65)	9.35	9.78	8.25	10.87	9.95 (.65)	9.67	10.23	8.56	10.98	4.0	4.0
	CL 400-m (Lap)	136.78 (20.46)	129.06	144.49	102.00	169.00	135.70 (17.65)	127.96	143.44	107.00	167.00	−0.8	0.85
	CL 1,160-m	362.11 (50.05)	343.23	380.99	277	460	359.55 (50.3)	337.51	381.59	273	450	−0.7	0.26
	SK 3,000-m parcours	630.71 (300.71)	517.28	744.14	487.9	741.9	655.99 (291.62)	534.12	777.85	527.40	791.5	4.0	0.21

CI-L/CI-U, confidence interval lower and upper bound 95%; SD, standard deviation; DP, double poling; V1 40-m, asymmetric 2:1; Diff. %, difference of girls vs. boys; XC, cross-country.

**Table 4:** Bonferroni–Holm adjusted correlations (*r*_xy_) and partial correlations (*r*_xy-z_: maturity offset as a confounder) between XC skiing test performance (seconds) and P_XC_.

	Boys (*n* = 36)		Girls (*n* = 22)		All (*n* = 58)
	*r*_xy_	*r*_xy-z_	*r*_xy_	*r*_xy-z_	*r*_xy_
*30–50-m sprint tests*					
V1 40-m uphill (s)	0.87***	0.89**	0.89***	0.82*	0.87***
DP 30-m uphill (s)	0.69***	0.69*	0.80***	0.81*	0.73***
Free skating 50-m (s)	0.85***	0.84**	0.88**	0.83**	0.85***
DP 50-m (s)	0.85***	0.75*	0.79***	0.89**	0.82***
Leg skating 50-m (s)	0.77***	0.74*	0.64**	0.52	0.70***

V1 40-m, asymmetric 2:1; DP, double poling; XC, cross-country; P_XC_, XC skiing performance index.

^*^ *p* < 0.05. ^**^p < 0.01. ^***^p < 0.001.

Girl's DP 30-m uphill (*r* = 0.80, *p* < 0.001) and boy's DP 50-m also showed a high loading (*r* = 0.85, *p* < 0.001).

Moderate correlations were found for boy's leg skating 50-m (*r* = 0.77, *p* < 0.001) and girl's DP 50-m (*r* = 0.79, *p* < 0.001) and P_XC_, while boy's DP 30-m uphill (*r* = 0.69, *p* < 0.001) and girl's leg skating 50-m (*r* = 0.64, *p* < 0.01) were acceptably related to P_XC_.

In boys, with maturity offset taken into account, V1 40-m uphill and free skating 50-m have a significantly high correlation with P_XC_ (*r*_xy-z _= 0.84 to 0.89, all *p*'s < 0.001). Similarly, leg skating 50-m and DP 50-m test performances have significant moderate correlations with P_XC_ (*r*_xy-z _= 0.74–0.75, all *p*’s < 0.05). When controlled by maturity offset, all XC skiing tests except for leg skating 50-m in girls are related to P_XC_ (*r*_xy-z_ = 0.71–0.89, all *p*’s < 0.05 to < 0.01) (Table 4).

### Multiple stepwise regressions

Multiple stepwise regression analyses with anthropometric and age-related variables, including the 1RM lat pull-down test and its percentage of weight results and XC-specific sprint tests, explain the following predicting models for the XC skiing performance of all, boys, and girls:

All:

$$P_{\mathrm{XC}}=0.401(V1-40\;m\;[s])+0.313\;(\mathrm{DP}\;50\;m\;[s])-8.187$$

$R^{2}=0.756\;(\;p<0.001),\;SEE=0.386$

Boys:

$$P_{\mathrm{XC}}=0.513(V1-40\;m\;[s])-5.855$$

$R^{2}=0.622\;(\;p<0.01),\;SEE=0.410$

Girls:

$$P_{\mathrm{XC}}=1,308(\mathrm{Free}\;50\;m\;[s])-12,392$$

$R^{2}=0.762\;(\;p<0.001),\;SEE=0.443$

### Sex differences

With the exception of the sum of skinfolds, no differences between girls' and boys' anthropometrics or calendar age were found. Boys' APHV was later (*p* < 0.001), leading to a negative maturity offset compared with a positive in the girls (*p* < 0.001) (Table 1). All other measurements of skiing time and speed did not show any variance between sexes. Differences (*p* < 0.05) have been found in the lat pull-down test (percentage of body weight) between boys and girls (Table 2).

## Discussion

The current study found moderate to high connections between short-duration maximal skiing speed (such as V1 40-m uphill, DP 30-m uphill, free skating 50-m, DP 50-m, and leg skating 50-m) and P_XC_ for all participants. Furthermore, the best indicators of P_XC_ for boys and girls were V1 40-m uphill, free skating 50-m, and DP 50-m when using stepwise regression models for predicting P_XC_. Additionally, maturity offset and age had acceptable to high correlations with P_XC_.

### Short-duration skiing tests (sprints)

#### Short-duration maximal speed tests

The study found that short-duration maximal speed in various forms of XC skiing, such as V1 40-m uphill, DP 30-m (uphill), and DP 50-m (flat), and free-and leg skating, were related to P_XC_ for both young boys and girls. For boys, this connection was found even when maturity offset was used to account for the effects of maturation, except for DP 30-m (uphill *r* = 0.69 ns). This result aligns with previous studies on adult skiers (18–19, 21, 33–36). When accounting for maturation in girls by using maturity offset, most short-duration tests still had high correlations with P_XC_, except for leg skating 50-m. Furthermore, the study findings regarding 50-m sprints align with the youth roller skiing study of Stöggl et al. (11), revealing that short-duration maximal roller skiing speed over 50 m in DP, V2 skating, and leg skating was related to P_XC_ for both young girls and boys, which is consistent with several studies on adult XC skiers (18–19, 21, 33–36).

Previous research by Stöggl et al. (18, 19) has shown that short-duration maximal speed in DP (treadmill) can predict performance in a DP sprint over a 1,000-m race distance in elite adult XC skiers. Additionally, short-duration maximal speed in DP and diagonal stride were found to have a strong correlation with performance in a classical-style sprint simulation on a treadmill over 1,100 m (18). Sandbakk et al. (21) found that peak speed during a short-duration incremental test was higher in male world-class sprint skiers compared to that in the national level ones using the V2 technique, but no differences were found in peak acceleration (30-m skating sprint) and maximal strength. Andersson et al. (18) found that maximal speed in DP and V2 skating was positively related to the percentage of racing time using the V2 technique during a sprint race. Similarly to the results found in elite XC skiers, the study finds that short-duration on-snow maximal speed is a time-efficient and highly predictive test of P_XC_ in young XC skiers, independent of age and sex. This result also highlights the importance of focusing on developing maximal speed abilities in youth XC skiers.

#### 50-m DP tests (flat)

The current study found that DP skiing performances over short distances were among the highest predictors of P_XC_ in youth XC skiers, independent of sex. This result aligns with a previous study on roller skiing and P_XC_ by Stöggl et al. (11). Mikkola et al. (20) examined the factors that predict performance in a simulated XC skiing sprint competition (4 m × 850 m) in elite male skiers on roller skis using the V2 skating technique on an indoor track and a 2-km × 2-km DP test. The study found that the 2-km × 2-km DP test was the best single performance predictor for sprint performance, indicating that sprint skiers should focus on sport-specific upper body training and training skiing economy at high speeds. Fabre et al. (37) found that peak speed during an incremental DP test in female elite XC skiers (4% grade test duration of 7–8 min) was related to competition performance (Italian Ski Federation Points). Moreover, Stöggl et al. (11) found that the girls' and boys' DP225-m uphill performances were also strongly related to P_XC_.

### Additional tests

#### 1RM lat pull-down test

The current study found that only girls' 1RM lat pull-down test had a low correlation with XC skiing performance. This finding is not in line with previous research on the association between strength and XC skiing performance in adult elite athletes. For example, Mikkola et al. (20) found that faster XC skiers had higher maximum and explosive force in a bench press test, a conclusion that aligns with a study by Stöggl et al. (38), which showed that power output during submaximal bench pressing and pulling was related to classical-style maximum speeds. Additionally, Losnegard et al. (39) found a strong correlation between one-repetition maximum on a seated pull-down exercise, simulating a DP pull and performance on a DP ergometer, time trial performance over 1.1-km double poling, and 1.3-km uphill skating performance.

Our research group recently found that a push-up test was related to XC skiing performance for both boys and girls. Additionally, boys' pull-up performance had an acceptable correlation with XC skiing performance. It is worth noting here that both tests measured muscular endurance power (strength perseverance) more than the maximal power (10) measured in the present 1RM lat pull-down test. So, it can be assumed that the crucial determinant of youth P_XC_ is not maximal power, but, instead, it is the skills necessary for cross-country skiing, which includes utilizing poles correctly, maintaining balance and posture, and maneuvering through different types of terrain.

### Sex differences

Except for the sum of skinfolds, there were no differences in anthropometrics or calendar age between girls and boys. Boys had a later PHV, which led to a negative maturity offset compared to a positive one in the girls.

It is worth noting that there were no significant differences in performance between boys and girls in different XC skiing tests and competitions. This finding contrasts with the previous study of roller skiing sprint, which showed significant differences for 50-m leg skating, V2 skating, and 50-m DP on roller skis, with better values for boys compared with girls (11).

## Strengths and weaknesses

Notable is the large sample of young cross-country skiers and their motivation during tests and competitions. Weaknesses concern the wide age group and the impact of growth and organic development on performance.

Similarly, the present and upcoming test results can hardly be replicated and compared due to varying snow and weather conditions at different test points in time.

## Limitations

Particularly among boys and girls, there is a strong correlation between XC performance and the measures of chronological age and maturity offset. Hence, it is crucial to account for maturation when testing this age group to ensure that the outcomes accurately reflect the intended measurements of physiological and technical abilities.

## Implications for future research

Observations of XC skiing careers over a longer period of time would be interesting for future investigations. Of course, the high dropout rate in elite youth sports is a challenge for this. Achieving this goal would require starting with a very large sample size and taking proactive measures to minimize the dropout rate.

Furthermore, the prognostic function of the collected data needs to be evaluated. The question of “How indicative are the performances achieved in youth years for the elite adult level?” need to be answered.

## Practical applications for athletes and coaches

The test concepts presented should provide practicable support for athletes and their coaches by measuring short-duration XCS sprint abilities. Furthermore, the comparison between individual athletes provides inspiration and motivation to push oneself and improve.

## Conclusion

In this study, it was found that the best indicators to determine P_XC_ in girls and boys were their performances in short-distance speed tests on snow, such as the 40 m (V1 up) to 50 m (flat) test, done using different techniques like double poling, free skating, or leg skating. These results align with previous studies on adult XC skiing, which also highlighted the importance of maximum speed, upper body strength, and endurance in determining performance, with DP performance being particularly significant.

The results showed that even when adjusting for the difference in maturity levels, the P_XC_ values for boys and girls were still closely linked to their performance in short-distance XC skiing sprints. This suggests that these sprint skiing tests are reliable and valid measures for predicting the P_XC_ of young people on snow.

In the context of the increases in skiing speeds and changes in skiing techniques on an elite level, it is also important that practice time must be focused early on in children and youth XC skiers to develop a wide range of motor abilities in addition to the above-mentioned ABCs. This might also include cardiovascular power, muscular endurance, and flexibility, as well as the specific technique and skills required for XC skiing such as the proper use of poles, balance and posture, and the ability to maneuver on varying terrains.

Therefore, coaches and trainers must take into account the unique developmental needs and abilities of young athletes when designing and implementing training programs. They should also be aware of the potential for natural growth and maturation to affect performance and use appropriate testing and evaluation methods to track progress and adjust the training program as necessary. Long-term monitoring and adaptations in training can help to distinguish the effects of the training from the effects of maturation.

## Data Availability

The raw data supporting the conclusions of this article will be made available by the authors, without undue reservation.
